# Evaluation of 6 and 10 Year-Old Child Human Body Models in Emergency Events

**DOI:** 10.1371/journal.pone.0170377

**Published:** 2017-01-18

**Authors:** Laure-Lise Gras, Isabelle Stockman, Karin Brolin

**Affiliations:** 1 Department of Applied Mechanics, Division of Vehicle Safety, Chalmers University of Technology, Gothenburg, Sweden; 2 Univ Lyon, Université Claude Bernard Lyon 1, IFSTTAR, LBMC UMR_T9406, Lyon, France; Universite de Nantes, FRANCE

## Abstract

Emergency events can influence a child’s kinematics prior to a car-crash, and thus its interaction with the restraint system. Numerical Human Body Models (HBMs) can help understand the behaviour of children in emergency events. The kinematic responses of two child HBMs–MADYMO 6 and 10 year-old models–were evaluated and compared with child volunteers’ data during emergency events–braking and steering–with a focus on the forehead and sternum displacements. The response of the 6 year-old HBM was similar to the response of the 10 year-old HBM, however both models had a different response compared with the volunteers. The forward and lateral displacements were within the range of volunteer data up to approximately 0.3 s; but then, the HBMs head and sternum moved significantly downwards, while the volunteers experienced smaller displacement and tended to come back to their initial posture. Therefore, these HBMs, originally intended for crash simulations, are not too stiff and could be able to reproduce properly emergency events thanks, for instance, to postural control.

## Introduction

The protection of children in motor vehicle crashes has improved with the use of child restraint systems; however, car crashes remain the second leading cause of death for children between 5 and 14 years old [1]. The head is the most frequently injured body region among forward-facing children [2,3]. To understand how children are injured, the causation scenarios of head injuries in frontal impacts for rear-seated, restrained children have been studied [4]. It was concluded that contact with the car interior is the principal cause of head injuries. In addition, emergency events such as braking, steering, or a combination of both influenced the kinematics of children prior to the impact, thereby affecting the child’s interaction with the restraint systems. Consequently, there is a need to evaluate child protection, during emergency events, together with the restraint systems and to understand the kinematic responses of children in pre-crash situations. A validated child numerical model is an attractive tool for the assessment of restraint systems in emergency events.

Numerical Human Body Models (HBMs) are increasingly used to simulate both pre-crash and in-crash occupant’s responses, especially adult models [5–10]. Nevertheless, child occupant models remain sparse and not all age groups are represented [11]. Finite Element (FE) models of children mostly represent the 3-year-old child [12–15]. Only the 3 year-old model presented by Mizuno et al. [13] has been successively improved to increase its biofidelity [14,15]. It has been validated by comparing its response with experimental corridors of child volunteer data for the abdomen (lap belt loading) and with the Hybrid III 3 year-old physical anthropomorphic test device response in calibration tests for the neck (pendulum test on the thorax, acceleration: 230 m.s^-2^ during 20 ms), thorax (pendulum test on the thorax, velocity: 6 m.s^-1^) and spine (flexion test at 45 degrees) [13–15]. However this model was excluded from the present study because it does not belong to the age group of interest, forward facing children aged 5 to 14 years. Okamoto et al. [16] presented an FE model of a 6 year-old pedestrian, although a publication presenting validation was not found. Two projects aim to develop FE models of children of different age groups: the Digital Child Project proposed by the Southern Consortium for Injury Biomechanics [17] and the Child Advanced Safety Project for European Roads [18]. Nevertheless, publications describing the development, validation, and application of these models were not found in the literature. Hence, there is a lack of available, validated, and well published child occupant FE models. Multi Body (MB) models of child occupants were presented by van Rooij et al. [19]. A 3 year-old child facet model was developed and extended to 6 and 10 year-old child HBMs in the MAthematical DYnamic MOdel (MADYMO) code (TASS International, Helmond, Netherlands) [20]. The anthropometry was based on the CANDAT database [21] and mechanical properties were scaled down from the 50^th^ percentile male model [20]. Validation of the 6 and 10 year-old models was presented in the MADYMO Human Models Manual [20]. Their response was compared with scaled corridors obtained from the dynamic hub impactor tests of Neathery [22] (impactor speed: 4.3 m.s^-1^ and 6.7 m.s^-1^). The 6 year-old model was also validated against: frontal thoracic pendulum tests (impactor mass: 3.5 kg, impactor speed: 6 m.s^-1^) performed on paediatric post mortem human subjects [23]; abdominal belt loading tests on porcine specimens [24]; and quasi-static neck tension tests on paediatric post mortem human subjects [25].

To the best of the authors’ knowledge, the published validations and applications of the child models have focused on the evaluation of child restraint systems or injury mitigation systems at acceleration levels above 20 g. Pre-crash or emergency events have not yet been studied in the literature with child numerical models, even though these manoeuvers have the potential to significantly influence the outcome of a car-crash. In pre-crash simulations the focus is on occupant kinematic instead of injury. Because of this, and in combination with the long duration of pre-crash events and the lack of suitable FE models, MB models were preferred in this study. In order to match the age group of interest, the 6 and 10 year-old commercially available MB models were chosen.

The aim of this paper was to perform a first comparison of the MADYMO 6 and 10 year-old child HBMs with experimental data from emergency events with child volunteers in order to highlight their strengths and weaknesses to reproduce such situations. Indeed, because these models were developed to be used in crash situations, and because of their lack of postural control compared with child volunteers, their response in emergency events may be not representative of child volunteers’ response. Therefore, knowing kinematic responses of the available models is an important first step towards their further development and validation in the pre-crash loading regime.

## Materials and Methods

The MADYMO HBMs representing 6 year-old (Child6YO) and 10 year-old (Child10YO) children [20] were used to simulate experiments previously performed with child volunteers in braking [26] and steering events [27]. The MADYMO Release 7.4.1 code was used for all simulations and post processing was done using MATLAB version R2010b (MathWorks, Natick, Massachusetts, U.S.A.).

### Experimental Data

Previously published studies have presented child volunteer data for braking [26] and steering [27] events performed on a test track with a Volvo XC70. The study protocol the authors are referring to was reviewed and approved by The Ethics Board of Gothenburg, Sweden [26,27]. The driving study was conducted on a closed-circuit test track by a professional driving instructor. Child volunteers were safely restrained and accompanied by a parent. Every child and parent were aware of the test protocol. An informed consent form was signed by the parents, and a small gift was given to the participating children. The test could be aborted at any time by the child or parent. Children were not exposed to any injury risks or major discomfort. The aims of this study were to quantify the kinematics of child occupants. A short summary is given here; please refer to the original publications for detailed information. Child volunteers were divided into two groups according to their stature: a short group (117±6 cm) and a tall group (143±5 cm). These stature requirements are close to the mean stature of 6 year-old (117 cm) and 10 year-old children (139 cm) [28]. All volunteers were restrained on the right rear seat of the vehicle. The short children were tested while seated on a booster cushion and the tall children were tested while seated on a booster cushion and while seated directly on the vehicle seat. All volunteers were restrained by the standard three-point seat belt of the test vehicle.

**Fig 1:**
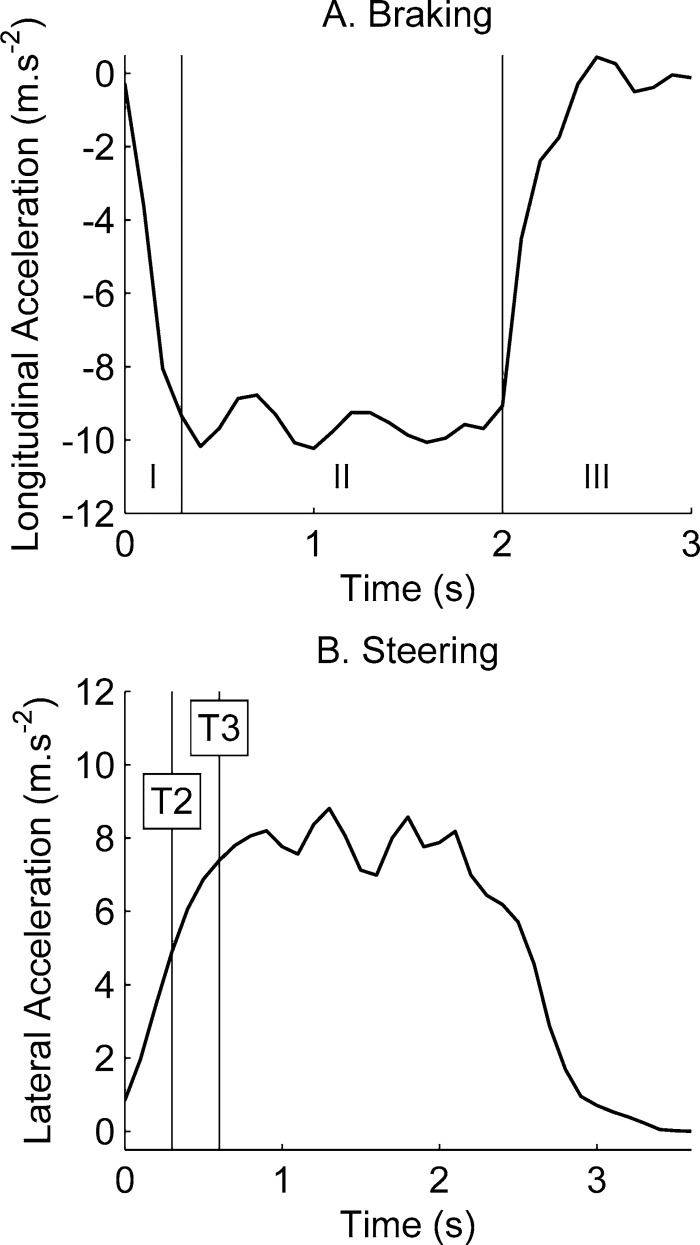
Acceleration as function of time for the two events, adapted from Stockman et al. **[26] and Bohman et al. [27]** with permission of the authors. (A) The braking event was split into three phases. (B) The steering event was studied at two points in time, T2 and T3.

During the braking event, the car was driven at 70 km/h and the driver applied the brakes as fast as possible to a full stop, resulting in a mean maximum longitudinal deceleration of 1 g. The braking event was divided into three phases (Fig 1). The first phase corresponded to a decrease of the longitudinal acceleration until reaching a plateau. The acceleration level was maintained during the second phase, and the third phase was the end of the event with the increase of the acceleration. Phase I finished at 0.3 s, and Phase II at 2 s.

The steering event was performed at 50 km/h with a mean maximum lateral acceleration of 0.8 g. The vehicle was turned to the right resulting in an inboard motion of the volunteers. The steering event pulse (Fig 1) presented the same shape as the braking event, but at a lower plateau value and with a longer duration. Data was presented for three points in time. A reference point T1, before the event started corresponded to the initial position of the volunteer. The point T2 was chosen in the middle of the ramping phase of the acceleration, 0.2 s after reaching 0.2 g, which corresponded to the time point 0.3 s in Fig 1. The point T3 was chosen 0.3 s after T2, i.e., at 0.6 s in Fig 1, and corresponded to the end of the ramping phase.

Volunteer kinematic responses were determined from video tracking of markers on the children. During steering events, kinematic analysis focused on the child’s lateral movement of the upper torso and seat belt position relative to the child’s shoulder, and during braking events, it focused on the forward trajectories for the forehead and external auditory canal (ear) as well as head rotation and shoulder belt force.

### Computational Models

#### Child HBMs

The Child6YO and Child10YO models are referenced in MADYMO as the child facet occupant models [19,20]. They are modelled with 92 bodies. In the spine, each vertebra is a separate body connected to neighbouring vertebrae by free joints with lumped joint resistance models. The thorax and abdomen are composed of flexible bodies and the pelvis is represented by facet elements. The shoulders, as well as the lower and upper limbs are a combination of rigid bodies and joints. The skin is represented by an FE mesh. In terms of anthropometry, the Child6YO model has a stature of 116 cm and weighs 21 kg, while the Child10YO is 144 cm tall and weighs 35.5 kg. These values were close to the volunteers’ anthropometry data [26]: children of the short and tall groups were respectively 117±6 cm and 144±5 cm tall and weighed 20 kg and 36 kg in average. Most of the mechanical properties are scaled down from the adult 50^th^ percentile male model and details can be found in the MADYMO Human Models Manual [20].

#### Seat, booster cushion and seat belt models

**Fig 2:**
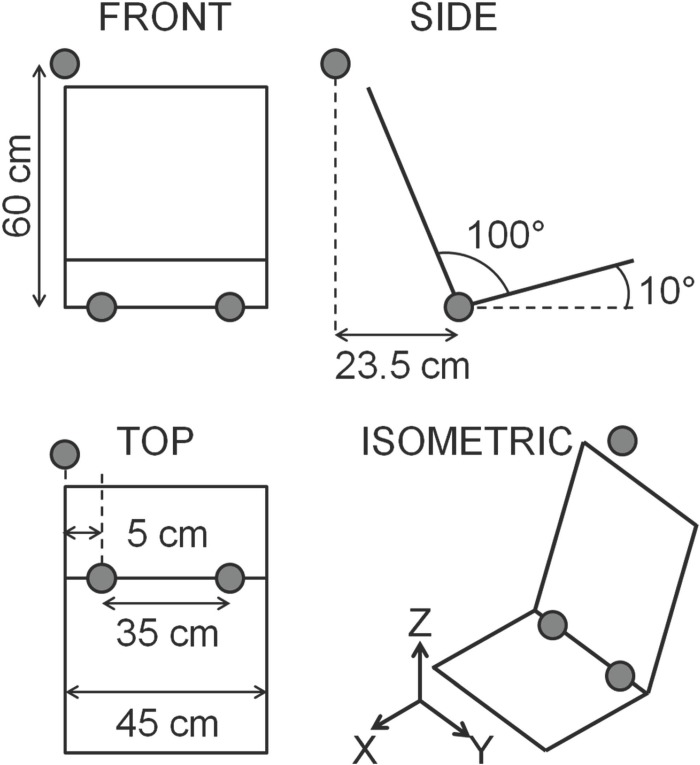
Scheme of the seat model. Belt anchorage positions are represented with grey dots. The global coordinate system is added to the figure.

The Child6YO model was simulated while seated on a booster cushion (Britax Ranger) model, previously published by Andersson et al. [29]. The Child10YO model was simulated while seated on the same booster cushion and while seated directly on the seat cushion. The seat model (Fig 2) was composed of two planes positioned to reproduce the geometry of the rear seat. The seat cushion was a 42-by-45 cm plane that made a 10° angle with the car floor. The seat back was a 55-by-45 cm plane, and the angle between the seat cushion and the seat back was 100°. Multi body contacts were defined between the child models and the booster and the seat, respectively. The predefined contact stiffness in the child models was used, which is a non-linear stiffness with hysteresis [30]. Contact was defined with a friction parameter. Friction coefficient was set to 0.3 for contacts between child models and the booster cushion and the seat cushion, and to 0.55 for the contact between the booster cushion and the seat cushion.

The three-point seat belt was modelled by FE and MB elements and had the geometry of the seat belt in the right rear seat of the vehicle. The MB elements connected the belt to the belt anchorages, and the triangular membrane FE modelled the interaction between the belt and the model. The lap belt’s anchorages were placed at the junction of the seat back and seat cushion (5 cm from the seat cushion right and left sides; Fig 2). The shoulder belt’s lower anchorage was the same as the right lap belt’s anchorage, while the upper anchorage was placed 60 cm above and 23.5 cm behind the left side of the seat back-to-seat cushion junction (Fig 2). When a booster cushion was used, intermediate points attached under the guiding loops of the booster were added to guide the belt. The belt was modelled without retractor. Data to model the seat and belt were obtained from the geometrical model of the Volvo XC70 car. An FE contact was defined between the belt elements and facet elements of the child model, based on the predefined skin mechanical properties.

#### Child models positioning

The initial positions of the child models were established by positioning each model just above the seat or booster cushion and applying gravity for 4 seconds until the model was in contact with the seat back and seat or booster cushion. During the positioning, the spinal joints of the model were locked. The joint positions were recorded and exported to define the initial joint positions of the models in the subsequent simulations. The models were centred on the seat. After positioning, each model was restrained by the seat belt using the belt-fitting tool available in XMADGic (TASS International, Helmond, Netherlands). When seated on the booster cushion the shoulder belt was routed under the left guiding loop, corresponding to the inboard side in a vehicle. Both the shoulder and lap belts were routed along the skin facet elements. When seated on the booster cushion the shoulder belt was positioned on mid-shoulder for both models while it was close to the neck for the Child10YO model when seated directly on the seat.

#### Braking and steering simulations

In total six simulations were performed: a braking and a steering simulation with the Child6YO model seated on the booster cushion, a braking and a steering simulation with the Child10YO model seated on the booster cushion, and a braking and a steering simulation with the Child10YO model seated directly on the seat cushion. The steering simulation was designed to represent a steering event to the right with an inboard motion of the occupant in the right rear seat of the vehicle. For each simulation, the braking and steering pulses (Fig 1) were applied to the seat model and gravity was applied to the whole system. The global coordinate system was defined with the X-axis pointing forward in the longitudinal direction, Y-axis in the lateral direction, and Z-axis upward in the vertical direction (Fig 2).

### Data Analysis

#### Braking event

For the braking event, the displacement of the forehead and upper sternum in the XZ-plane of the global coordinate system was output. In order to compare the volunteer data with simulation data, the vertical displacements were plotted according to the seated height of the models and volunteers, and the longitudinal displacements were considered to start in the same point. The forehead point was defined as a node on the forehead above the eyes. The upper sternum point was a node on the skin, at the level of T1. A point defining the auditory canal was used to calculate the change of head rotation in the sagittal plane. This point was defined as the node at the right-most point of the head in the mid-sagittal plane of the head. The shoulder belt force was measured. Forehead displacement, head rotation and shoulder belt force were compared with volunteer data.

#### Steering event

For the steering event, the forehead and upper sternum displacement in the YZ-plane of the global coordinate system were measured at times T2 and T3 (defined in Fig 1). This corresponded to the inboard displacement relative to the centreline of the seat. Only the lateral displacement of the upper sternum was compared with volunteer data.

### Evaluation of Simulations’ Robustness with a Full Factorial Design of Experiments

In order to evaluate the robustness of the performed simulations, a two-levels full factorial design of experiments was performed [31]. The response variables were the displacements of the forehead and upper sternum at the end of phase I for the braking event and at T2 for the steering event. These response variables were considered depending on four parameters: child model initial posture (A), belt routing (B), contact friction coefficient between child and booster cushion and/or seat (C), and contact friction coefficient between booster cushion and seat cushion (D) (Table 1). The child model initial posture (A) was defined either as the child model leaning against the seat back (according to the chapter Child models positioning) or as the child model in a more upright position with an angle of 0.2 radians at the joint between the sacrum and the 5^th^ lumbar vertebra. The upright position was chosen to represent a child moving forward in order to reach something or speak with the driver. The belt routing parameter (B) corresponded either to the same seat belt anchorage as described in Fig 2 or to a seat belt anchorage with the upper anchorage of the shoulder belt placed 2 cm closer to the middle of the seat back along the Y direction that resulted in a shoulder belt position closer to the neck of the child models. These postures were chosen because they were representative of the shoulder belt initial position noticed during the experiments with child volunteers [27]. Contact friction coefficient between the child model and the booster cushion and/or seat (C) was set either to 0.2 or 0.4. These values were chosen arbitrarily around the value of 0.3 which was used in the main simulations. The contact friction coefficient between the booster cushion and the seat cushion (D) was set either to 0.3 or 0.8. These values were chosen around the value of 0.55 which was used in the main simulations, and were also chosen so that they were similar to friction coefficients in the parametric study with a 3-year-old multibody model in crash events by Andersson et al. [29].

**Table 1:** Parameters of the design of experiments, their levels for each model and associated number of simulations.

MODELS	6YO on booster cushion	10YO on booster cushion	10YO on seat cushion
PARAMETERS	PARAMETERS LEVELS		
Parameter A: Child model initial posture	Leaning against the seat back	Leaning against the seat back	Leaning against the seat back
	Upright position	Upright position	Upright position
Parameter B: Belt routing	Standard position	Standard position	Standard position
	Closer to the neck	Closer to the neck	Closer to the neck
Parameter C: Friction coefficient between the child model and the booster or seat	0.2	0.2	0.2
	0.4	0.4	0.4
Parameter D: Friction coefficient between the booster cushion and the seat	0.3	0.3	
	0.8	0.8	
NUMBER OF SIMULATIONS	16 braking	16 braking	8 braking
	16 steering	16 steering	8 steering

The effect of all the parameters (A-D) on the response variables were evaluated for each child model, and for both braking and steering events. This resulted in a total of 80 simulations (Table 1). To compute the effect of a parameter, the average response of all simulations for which the parameter was set at its first level was subtracted from the average response of all simulations in the considered model configuration, and the same calculation was done for the second level of each parameter. The trajectories of the forehead and upper sternum obtained for the 80 simulations were also compared with the results of the six main simulations.

## Results

### Braking Event

**Fig 3:**
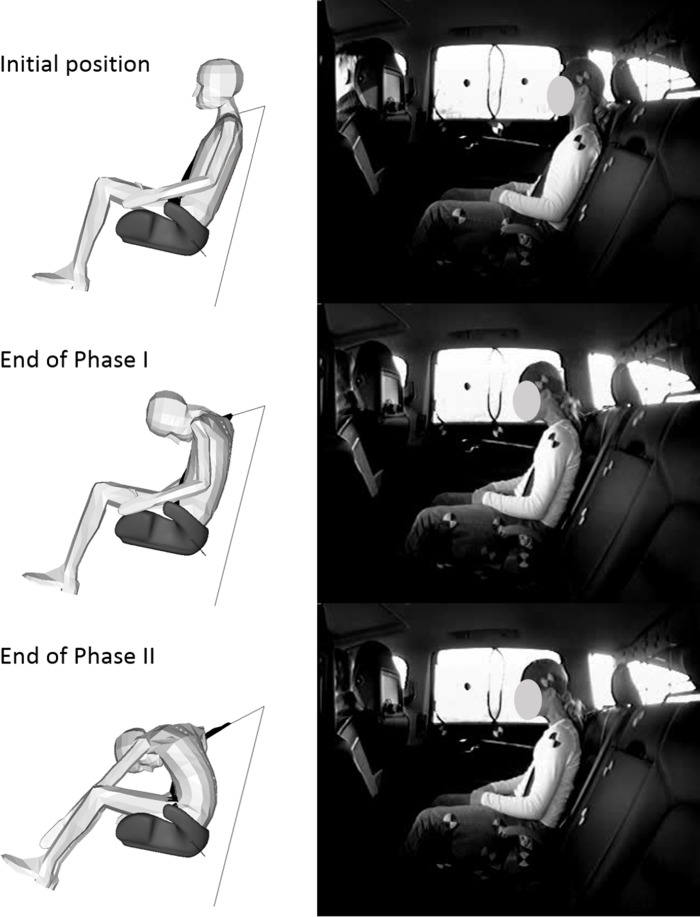
Simulation results for the Child10YO. The Child10YO response when seated on the booster cushion is compared with a child volunteer of corresponding age at three points in time during the braking event: initial position, end of Phase I (0.3 s) and end of Phase II (2 s). Figures extracted from the datasets presented in Stockman et al. [26] with permission of the authors.

**Fig 4:**
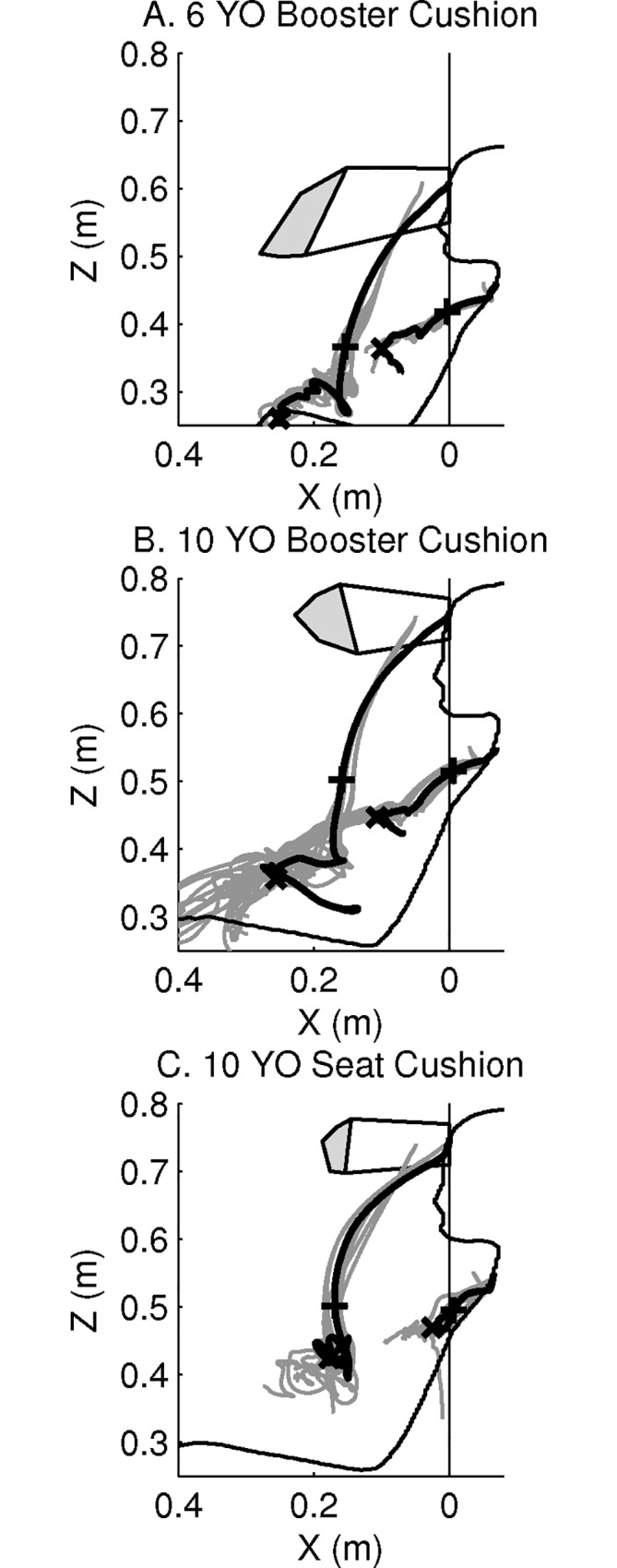
Forehead and upper sternum displacements in the braking event, in the XZ-plane of the global coordinate system. Displacements are presented for the Child6YO (A), the Child10YO on a booster cushion (B) and on the seat cushion (C), and the experimental data [26]. HBMs: black solid line. Corridor from volunteer data: white area with black solid border and peak displacements in grey area. Symbol +: end of Phase I. Symbol x: end of Phase II. Displacements obtained from the design of experiments until end of Phase II: grey solid lines. Contour of child HBM overlaid for reference. Experimental data used with permission of the authors.

The kinematic responses of the child HBMs were different from the child volunteers and did not fall within the experimental corridors of the volunteer data (Figs 3 and 4). During the simulations, the upper torso of the model started to move forward while the head moved forward and downward. Then, because of the interaction with the shoulder belt and the head’s inertia, a rotation of the upper torso around the vertical axis took place, especially when the models were seated on the booster cushion. As a consequence, for the models seated on the booster cushion, the belt slipped off the shoulder during the acceleration plateau of the braking pulse, and the head continued to move downward. Analysis of the shoulder belt position on the shoulder prior to the braking event and at maximum forward displacement, for the child volunteers, showed that the shoulder belt maintained the same position throughout the event for all children except two in the short group. One where the shoulder belt slipped off the shoulder momentarily before the braking event and one where the shoulder belt slipped off during the braking event, both when seated on a BC.

Generally, the HBMs experienced a greater forward displacement than the volunteers (Fig 4, Table 2). The forward displacements in the XZ-plane of the HBMs at the end of Phase I were 2–7 cm greater than the volunteers’ mean displacement. At the end of Phase II the difference was greater than 10 cm and outside the volunteer ranges for the HBMs on booster cushion, while the Child10YO on the seat cushion was within the experimental range. The displacement of the forehead and upper sternum targets were also different if a booster cushion was used (Fig 4). The forward displacement at the end of Phase II of the Child10YO model seated on a booster cushion was 44% greater for the forehead and 100% greater for the upper sternum than for the same model when seated directly on the seat cushion. The change of head rotation was within the range of volunteer data for Child6YO, while the head rotation for Child10YO was greater than volunteer data. The change of head rotation (30°) was the same for both models regardless of restraint system.

**Table 2:** Forehead and upper sternum target displacements relative to the initial position during the braking event.

[m]	FOREHEAD				UPPER STERNUM			
	End of Phase I		End of Phase II		End of Phase I		End of Phase II	
	X	Z	X	Z	X	Z	X	Z
Short Children BC	0.13	-0.02	0.13	-0.01	-	-	-	-
	min: 0.08	min: -0.04	min: -0.03	min: -0.07	-	-	-	-
	max: 0.21	max: -0.01	max: 0.22	max: 0.03	-	-	-	-
Child6YO BC	0.15	-0.24	0.25	-0.35	0.07	-0.04	0.17	-0.10
Tall Children BC	0.13	0.00	0.15	0.00	-	-	-	-
	min: 0.11	min: -0.02	min: 0.08	min: -0.05	-	-	-	-
	max: 0.15	max: 0.01	max: 0.21	max: 0.03	-	-	-	-
Child10YO BC	0.16	-0.25	0.26	-0.39	0.06	-0.03	0.18	-0.10
Tall Children SC	0.10	0.00	0.15	0.02	-	-	-	-
	min: 0.06	min: -0.01	min: 0.13	min: -0.02	-	-	-	-
	max: 0.13	max: 0.01	max: 0.19	max: 0.05	-	-	-	-
Child10YO SC	0.17	-0.25	0.18	-0.33	0.06	-0.05	0.09	-0.08

Forehead and upper target displacement at the end of Phase I and II during the braking event, for HBMs and child volunteers [26]. All values are expressed in meters. Data for child volunteers are mean values. Experimental data used with permission of the authors.

**Fig 5:**
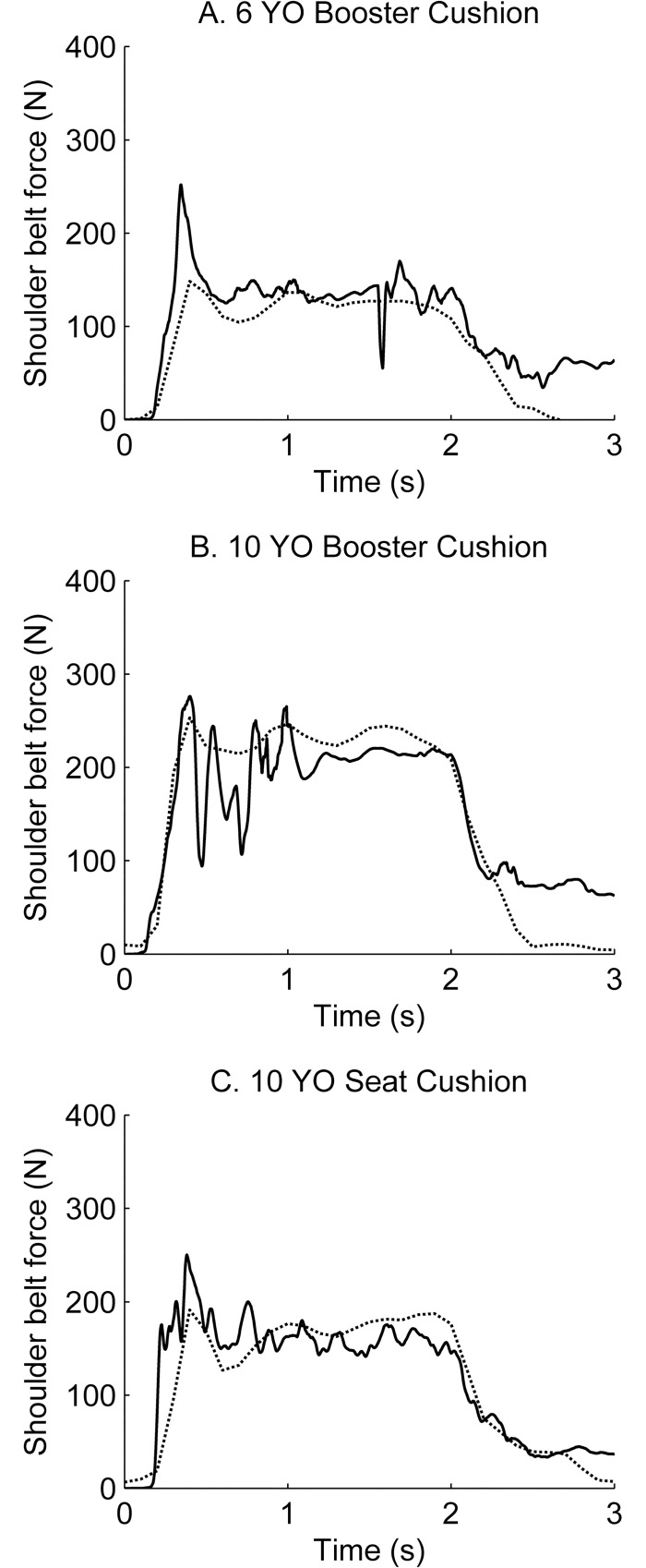
Low pass filtered shoulder belt force over time. Shoulder belt force are presented for the Child6YO (A), the Child10YO on a booster cushion (B) and on the seat cushion (C) and compared with experimental data extracted from the datasets presented in Stockman et al. [26] with permission of the authors. HBMs: black solid line, child volunteers: black dotted line.

The shoulder belt force was inside the range of volunteer data for all simulations (Fig 5). As mentioned previously, the belt slipped off the shoulder for the models when seated on the booster cushion. For the Child6YO model this happened at the end of Phase II (at 1.6 s), while the belt slipped off the shoulder in the beginning of Phase II (at 0.5 s) for the Child10YO model.

### Steering Event

**Fig 6:**
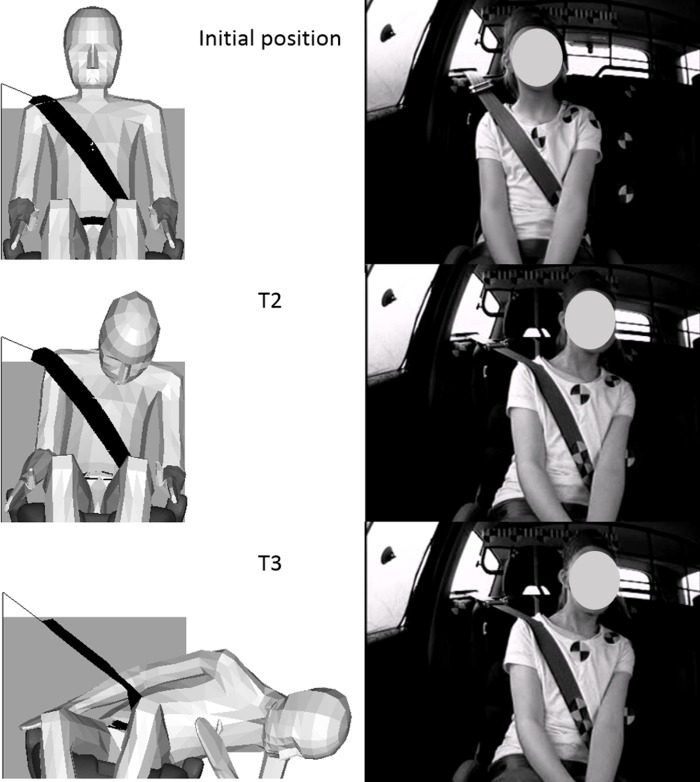
Simulation results for the Child10YO. The Child10YO response when seated on the booster cushion is compared with a child volunteer of corresponding age at three points in time during the steering event: initial position, T2 (0.3 s) and T3 (0.6 s). Figures extracted from the datasets presented in Bohman et al. [27] with permission of the authors.

**Fig 7:**
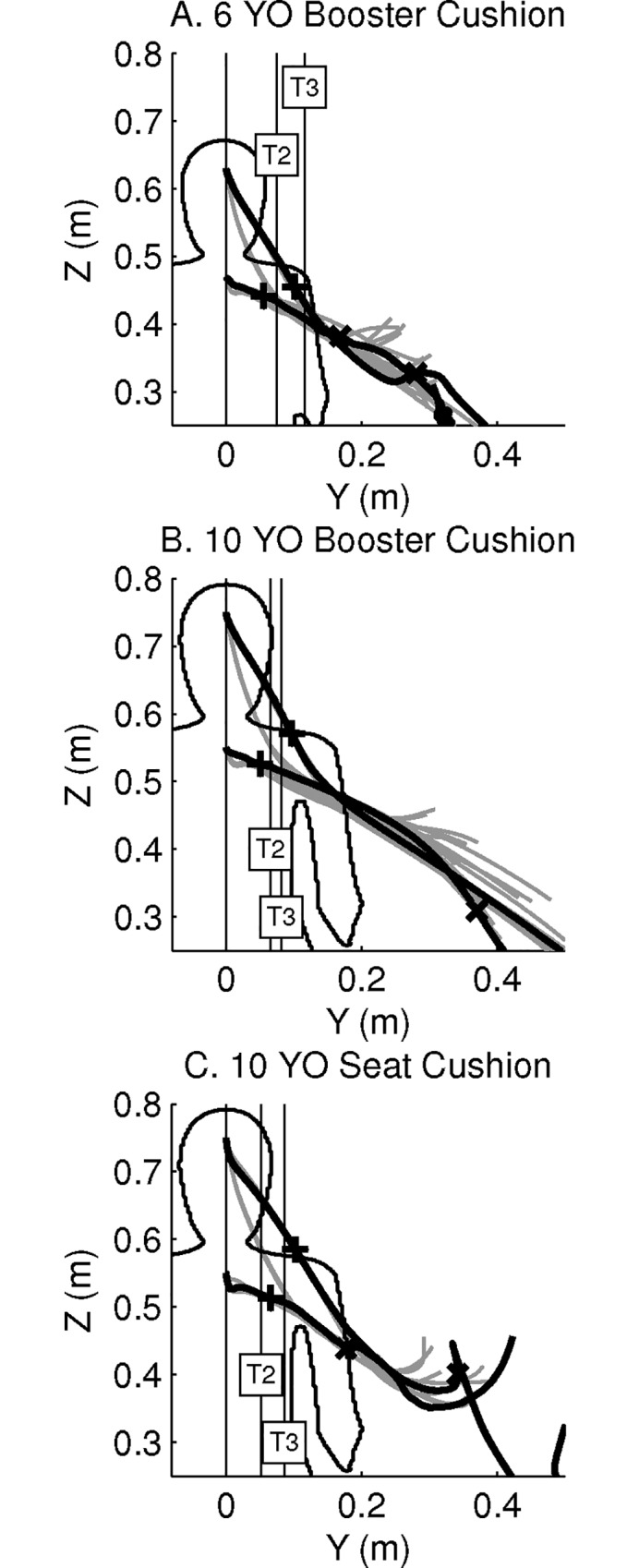
Forehead and upper sternum displacements in the steering event, in the YZ-plane of the global coordinate system. Displacements are presented for the Child6YO (A), the Child10YO on a booster cushion (B) and on the seat cushion (C), and the experimental data [27]. HBMs: black solid lines. Symbol +: time T2. Symbol x: time T3. Vertical lines labelled T2 and T3: volunteers’ data for the upper sternum. Displacements obtained from the design of experiments until T3: grey solid lines. Contour of child HBMs overlaid for reference. Experimental data used with permission of the authors.

In the steering event, as for the braking event, the child HBMs moved differently than the child volunteers (Figs 6 and 7). At the beginning of the simulation, the head and upper torso moved laterally (along the Y-axis) until the lower part of the torso was stopped by the belt or the guiding loop of the booster cushion. At the same time, the head was rotating around the Y-axis until the chin reached the chest which implied a vertical downward displacement of the forehead. Then lateral bending of the upper torso appeared, implying a lateral and downward displacement of the head. For all the simulations, the models slipped out of the shoulder belt.

In the YZ-plane, the lateral and vertical displacements of the forehead and upper sternum are presented in Fig 7 and Table 3. At T2, the HBMs had a lateral displacement of the upper sternum within the range of experimental data. However at T3, all simulations resulted in 1.7–4.6 times greater lateral displacement than the mean values for the different groups of volunteers. The lateral displacement of the Child10YO model seated on a booster cushion was 59% greater for the forehead and 105% greater for the upper sternum than for the same model when seated on the seat cushion.

**Table 3:** Forehead and upper sternum displacement relative to the initial position, at T2 and T3 during the steering event.

[m]	FOREHEAD				UPPER STERNUM			
	T2		T3		T2		T3	
	Y	Z	Y	Z	Y	Z	Y	Z
Short Children BC	-	-	-	-	0.07	-	0.10	-
	-	-	-	-	min: 0.03	-	min: 0.06	-
	-	-	-	-	max: 0.15	-	max: 0.17	-
Child6YO BC	0.10	-0.17	0.28	-0.30	0.05	-0.03	0.17	-0.09
Tall Children BC	-	-	-	-	0.07	-	0.08	-
	-	-	-	-	min: 0.03	-	min: 0.06	-
	-	-	-	-	max: 0.10	-	max: 0.10	-
Child10YO BC	0.10	-0.18	0.54	-0.54	0.05	-0.02	0.37	-0.24
Tall children SC	-	-	-	-	0.07	-	0.10	-
	-	-	-	-	min: 0.04	-	min: 0.06	-
	-	-	-	-	max: 0.11	-	max: 0.13	-
Child10YO SC	0.10	-0.16	0.34	-0.35	0.07	-0.04	0.18	-0.11

Forehead and upper sternum displacement relative to the initial position, at T2 and T3 during the steering event, for HBMs and child volunteers [27]. All values are expressed in meters. Data for child volunteers are mean values. Experimental data used with permission of the authors.

For all HBMs, the forehead and upper sternum had a downward vertical displacement of about 0.17 m and 0.03 m at T2 respectively and up to 0.54 m and 0.24 m at T3 respectively (Table 3), whereas a qualitative analysis of the video data from the volunteer tests showed that child volunteers’ upper body had almost no downward displacement.

### Full Factorial Design of Experiments

**Fig 8:**
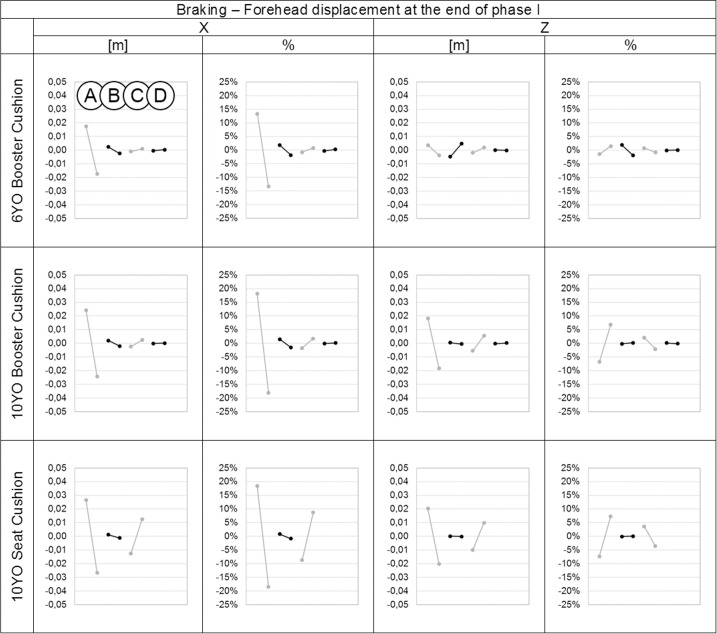
Effect of the parameters on the forehead displacements in the X and Z directions in meters and percentage for each child model at the end of phase I during the braking event. On each graph from left to right: Parameter A: Child initial posture, Parameter B: Belt routing, Parameter C: Contact friction coefficient between child model and booster and/or seat cushion. Parameter D: Contact friction coefficient between booster cushion and seat cushion.

**Fig 9:**
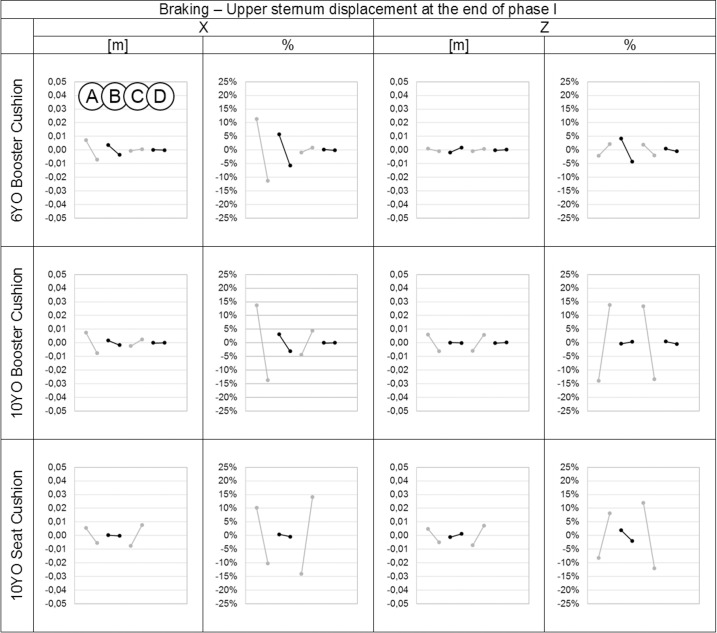
Effect of the parameters on the upper sternum displacements in the X and Z directions in meters and percentage for each child model at the end of phase I during the braking event. On each graph from left to right: Parameter A: Child initial posture, Parameter B: Belt routing, Parameter C: Contact friction coefficient between child model and booster and/or seat cushion. Parameter D: Contact friction coefficient between booster cushion and seat cushion.

**Fig 10:**
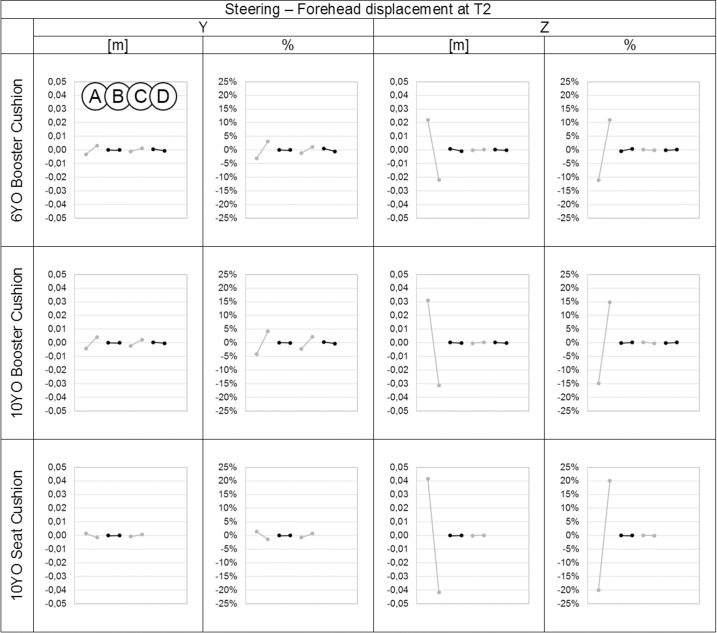
Effect of the parameters on the forehead displacements in the Y and Z directions in meters and percentage for each child model at T2 during the steering event. On each graph from left to right: Parameter A: Child initial posture, Parameter B: Belt routing, Parameter C: Contact friction coefficient between child model and booster and/or seat cushion. Parameter D: Contact friction coefficient between booster cushion and seat cushion.

**Fig 11:**
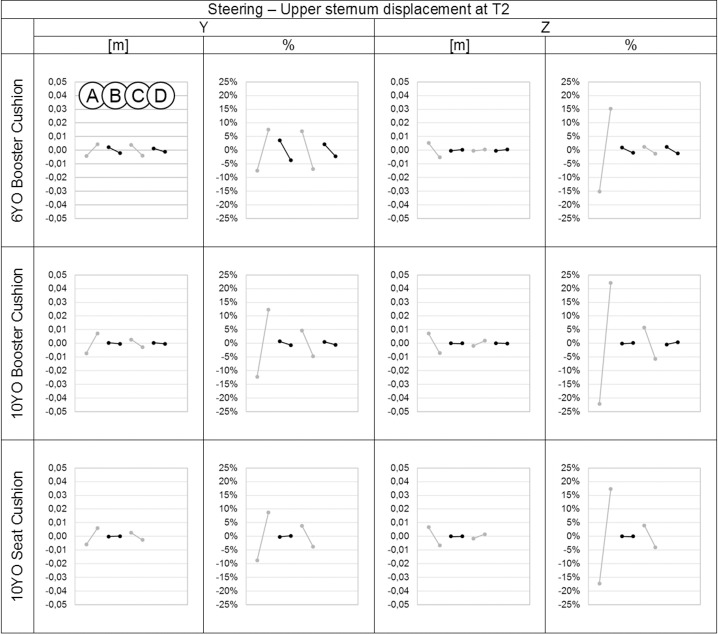
Effect of the parameters on the upper sternum displacements in the Y and Z directions in meters and percentage for each child model at T2 during the steering event. On each graph from left to right: Parameter A: Child initial posture, Parameter B: Belt routing, Parameter C: Contact friction coefficient between child model and booster and/or seat cushion. Parameter D: Contact friction coefficient between booster cushion and seat cushion.

The trajectories of the forehead and upper sternum for each simulation of the design of experiments are plotted in Fig 4 for the braking event and in Fig 7 for the steering event. Results are close to the trajectories of the six main simulations, especially during the first phase of each event. The analysis of the design of experiments showed that parameter A was the parameter that had the largest influence on the kinematics results for both events (Figs 8, 9, 10 and 11).

The initial child model position (parameter A) had the largest effect on forehead and upper sternum displacements in both X and Z directions (up to 18%) for braking and in both Y and Z directions (up to 22%) for steering. This corresponds to a maximum variation of 4 cm for the displacement of forehead and 1 cm for the upper sternum. The contact friction coefficient between the child model and the booster cushion and/or seat (parameter C) had a lower effect on the forehead and upper sternum displacements (up to 15%), that corresponded to a maximum variation of 1.3 cm for the forehead and of 0.8 cm for the upper sternum. Regarding the other parameters (B and D), belt routing had a marginal effect on the results, and the contact friction coefficient between the booster cushion and seat cushion had almost no effect on the results in either braking or steering events.

## Discussion

The aim of this study was to evaluate the kinematic responses of 6 and 10 year-old child numerical models in emergency events. This was done by comparing the responses of the Child6YO and Child10YO models available in MADYMO with child volunteer data in braking and steering events. To the best of the authors’ knowledge, this is the first study to present the responses of child HBMs in low acceleration loadings.

The HBM forward and lateral displacements were within the range of volunteer data in the beginning of the braking and steering events, up to approximately 0.3 s. Later in the events, the HBMs had greater displacements than the volunteers in all cases where the seat belt had slipped of the shoulder. The only case without belt slip off was the Child10YO on the seat cushion in the braking event, where the model response at 2 s was only 3 cm greater than the volunteers mean displacement, still within the ranges. However, the downward motion of the HBMs’ sternum and head is not representative of the kinematic response of child volunteers. This results highlights the high flexibility of the models’ spine, which is an interesting characteristic. Indeed, since these child HBMs were developed for crash scenarios, they were expected to have a stiffer kinematic response, and thus a stiffer spine than child volunteers.

Peak belt forces occurred before belt slip-off in all braking simulations and were relatively close to the mean experimental peak values of volunteer data. However because of the head and upper torso kinematics, the belt was sliding more on the HBMs’ shoulders than on the child volunteers. Since shoulder belt performance is a function of the position of the belt on the shoulder, improvements of the HBMs’ kinematic response are desirable to obtain a numerical tool suitable to study the loading of restraint systems, and thereby to evaluate and optimize restraint performance.

As mentioned, the HBMs slipped out of the shoulder belt during the steering event before 0.3 s, while the child volunteers, especially the tall ones, tended to move the shoulder up and forward in order to maintain the shoulder belt on the shoulder [27]. For short volunteers the belt had slipped off before 0.3 s in 7 out of 16 trials and before 0.6 s in 10 out of 16 trials. Based on visual inspection of the volunteer video data, it seems that they tried to maintain their head in an upright position and attempted to return to their initial seated posture. However, the HBMs’ heads had a downward motion coupled with head rotation and the models continued to move laterally and forward throughout the events. The volunteer kinematics and the interaction with the shoulder belt were influenced by muscle activity, which is not present in the HBMs and can explain some of the differences seen.

The change of head rotation in braking events was expected to be greater for the HBMs than for the volunteers due to the lack of muscle activity, and due to spine flexibility. This was true for the Child10YO model, but the response of the Child6YO model was within the range of the volunteers in the braking. The children were not given specific instructions regarding how to sit and behave in the vehicle, resulting in differences in the initial seated posture and head position among the test subjects. As a result, small differences in the position of the target point on the forehead at the start of the event could be seen. Therefore only normalised head rotations were calculated. This spread in the initial head rotation in the volunteer study [26] resulted in great variations among the volunteers, especially the short group, which is important to take into account when comparing the models with the volunteer data. It is worth noting that the change of head rotation was similar for all the HBMs and that the maximum head rotation was reached earlier than for the child volunteers. This result suggests that due to the absence of muscle activity, the HBMs’ head fell because of gravity loading rather than braking acceleration. This result was confirmed with a static simulation with only gravity loading.

The design of experiments highlighted that the kinematic responses of child HBMs were affected by the child model initial posture and the contact definitions between the child model and the booster cushion and/or seat cushion. However, this did not impose a major change to the global kinematics of the different HBMs. Therefore, it seems relevant to use the kinematic results obtained from the six main simulations to assess the HBMs performance. Nevertheless, in future work, these two parameters should be considered and measured to simulate child volunteer data. Regarding the other parameters, the belt routing had only a small effect on kinematic data and the contact friction coefficient between the booster cushion and seat had almost no effect on the results. This last result was in accordance with the work by Andersson et al. [29] who also evaluated this parameter but for a 3-year-old multibody model in crash events.

The discussion this far has highlighted that the HBMs behaved differently than the volunteers during the entire braking and steering events. To increase the biofidelity of these models, changes in their mechanical properties can be made. It is possible to increase the spine stiffness in both frontal and lateral bending. This could for instance increase the difference in change of head rotation between the Child6YO model and Child10YO model; and also compensate for the lack of muscle tension. Nevertheless, in order to reproduce the whole event, changing the spinal mechanical properties would not be enough as it would not prevent the model from falling due to gravity later in the events. At this point, the volunteers are most likely actively contracting their muscles in order to try to return to the initial seated posture. Increasing model stiffness alone will not be sufficient to achieve biofidelic kinematics throughout the duration of the events. Moreover, as mentioned previously, the child volunteers seemed to activate their muscles during these emergency events, especially the tall children. In the braking event, tall children managed to keep an upright posture by tensing their muscles, while it seemed more difficult for short children. During the steering events, tall children tended to rotate their upper body and lift their outboard shoulder in order to maintain contact with the shoulder belt, while short children were sliding inboard and tried to compensate by bracing themselves with their arms on the seat cushion or guiding loops of the booster cushion [27]. In these cases, passive HBMs would not be suitable to model the kinematic response of the children and the differences between short and tall children. Postural control is required to reproduce these behaviours with HBMs, and differences in muscle maturity must be taken into account.

Different approaches have previously been used to model active muscle response in adult HBMs and body segments. One approach is to include active muscles elements in the existing models, as done in neck models [32–35] with muscles activation defined as a function of time. Another approach is to include muscle elements with activation defined using closed loop control to maintain the body posture. This has for instance been done for the whole upper body [8] or for the lower body [36,37]. A similar approach has been implemented in the MADYMO 50^th^ percentile adult male HBM, but instead of controlling active muscles elements, torques were applied in the joints of the spine with closed loop control. This so called active spine has the ability to provide the model with postural stability to enable that it can return back toward its initial position [7,38]. It is suggested that implementation of such active response should be further investigated in order to design a biofidelic numerical tool relevant for modelling the response of 6 and 10 year-old forward-facing children during pre-crash events.

Recently, we took the first steps towards active child HBMs by implementing postural control in the Child6YO model at the spine level [39]. Torque actuators, sensors and controllers were implemented at each vertebral level for the three rotational degrees of freedom. The torque needed to stabilize the spine in each direction (flexion-extension, lateral bending, and axial rotation) was divided into two components: a static torque to compensate for gravity loadings, and a dynamic torque so that the model would strive to come back to its initial position if a disturbance was applied. A first set of control parameters were adapted from the literature on adults [8,38], reduced by half to represent a child response, and subsequently tuned so that the active child HBM kinematic response was within the range for child volunteers in the braking and steering events presented previously in this paper [26,27]. Finally, the model was validated with another set of steering event data and applied to study the influence of steering pulse shape on the inboard movement of children, which directly affect the shoulder belt performance in case of a crash.

To go further, the same set of controllers will be implemented in the Child10YO model. However, based on the results obtained with the passive models, we can assume that control parameters will be different than from the active Child6YO model. Indeed, both passive models had a similar kinematic response while short and tall volunteers behaved differently: tall volunteers showed a better postural control than short volunteers, especially during the braking events where they maintained an upright posture. Therefore, we expect control parameters for the active Child10YO model to be higher than for the active Child6YO model. Moreover, the Child10YO model was evaluated when seated on the booster cushion, and when seated directly on the seat which changed its interaction with the seat belt, and thus affected its passive response. Interaction with the seat belt may therefore also affect control parameters values, making it difficult to find one set of postural parameters usable for reproducing both sitting postures. An optimization method will be needed to address this issue. Finally, postural control in the Child10YO model may not be enough to reproduce tall volunteers’ kinematics. Several child volunteers in the tall group tried to raise their outboard shoulder to maintain contact with the seat belt, and thus inducing a torso rotation. Implementation of active muscle elements, like previously introduced may be useful to reproduce this behaviour.

As a conclusion, studying the passive response of these child HBMs gave us important insight in order to improve the Child6YO and Child10YO models and thus make them more biofidelic. Results with the active Child6YO model also illustrate the potential of active child HBM’s: to analyse child kinematics and the interaction with restraint systems, to study the risk of interior impacts considering the full sequence of the crash, and to study how the restraint design can influence the child’s posture and thereby the efficiency of the restraint system. Indeed, it has been highlighted in the literature that initial posture significantly affected the child kinematics in a crash [19].

There are several limitations with this study. The emergency events were simulated with multi body dynamic models of the child, the rear seat, and the booster cushion. The entire vehicle was not modelled and therefore, the pitch, roll and suspension effects were not taken into account and the pulse that was applied to the seat model was limited to the experimentally measured longitudinal or lateral vehicle acceleration, depending on the reproduced event. Another experimental study [40] concluded that vehicle pitch was low during braking events and had minor influence on the volunteer kinematics. Therefore, as a first approach, it appeared reasonable to focus only on the main loading experienced by the children: the longitudinal acceleration for braking, and lateral acceleration for steering.

The rear seat was simplified by two rigid surfaces, with any seat deformation caused by the child mass captured only by the contact stiffness, which might have had an effect on the global kinematics. Nevertheless, using rigid planes to model a car seat has been previously done [41,42]. Moreover, Johansson et al. [43] found marginal effects of the seat stiffness on the global head kinematics of a three-year old child model compared with the effect of the seat inclination, which in this study was the actual geometry of the test vehicle’s rear seat. Indentation test of front row seats by Östh et al. [8] give that a force of 355 N (Child10YO) produced less than 2 cm deformation of the softest part of the cushion. Since the mass of the child is distributed by the booster on a larger area the seat indentation for the child volunteers will be even smaller and it is not expected to influence the response. In the model, the seat back and seat cushion were considered flat with only one angle of inclination; these parts are slightly curved which can affect the spine curvature, and lead to differences between the volunteers and the models. However, the spine curvature for the HBMs was chosen in accordance with the recommendations provided by MADYMO.

The booster cushion model in the simulations was similar to the booster cushion used during the experiments, albeit slightly smaller. Compared with the experimental booster cushion, the guiding loops were approximately 7 cm closer to the back of the booster, the inclination was about 4° less, and the length and width were approximately 3 cm shorter. These differences might affect the belt position, although the global kinematics of the models was expected to remain the same. It is tempting to investigate the influence of these differences with several different models of booster cushions in a future study, this was not the purpose of the present study.

## Conclusions

The numerical kinematic response of two child HBMs representing 6 and 10 year-old children was less stiff compared with child volunteers in braking and steering events, especially in the later stages of the events. The models fell to the side in the steering event, while child volunteers tended to come back to their initial position. The belt slipped off the shoulder for almost all simulations, while it did not slip off the shoulder for all the child volunteers. However, before the shoulder belt slipped off, the forward and lateral displacements were within the volunteer ranges (greater than the mean value). As a conclusion, the strength of these child HBMs is that they are not too stiff as could have been expected of models developed for crash. Nevertheless, further developments are desirable to obtain child HBMs suitable for reproducing emergency events. Indeed, implementing postural control will give positive results for global kinematics of the models and their interactions with the belt.
